# Calcium Signaling during Cortical Apical Dendrite Initiation: A Role for Cajal-Retzius Neurons

**DOI:** 10.3390/ijms241612965

**Published:** 2023-08-19

**Authors:** Joshua R. Enck, Eric C. Olson

**Affiliations:** Department of Neuroscience and Physiology, State University of New York Upstate Medical University, 505 Irving Ave., Syracuse, NY 13210, USA; enckj@upstate.edu

**Keywords:** Cajal-Retzius neurons, cortical projection neurons, glutamate, glycine, dendritogenesis

## Abstract

The apical dendrite of a cortical projection neuron (CPN) is generated from the leading process of the migrating neuron as the neuron completes migration. This transformation occurs in the cortical marginal zone (MZ), a layer that contains the Cajal-Retzius neurons and their axonal projections. Cajal-Retzius neurons (CRNs) are well known for their critical role in secreting Reelin, a glycoprotein that controls dendritogenesis and cell positioning in many regions of the developing brain. In this study, we examine the possibility that CRNs in the MZ may provide additional signals to arriving CPNs, that may promote the maturation of CPNs and thus shape the development of the cortex. We use whole embryonic hemisphere explants and multiphoton microscopy to confirm that CRNs display intracellular calcium transients of <1-min duration and high amplitude during early corticogenesis. In contrast, developing CPNs do not show high-amplitude calcium transients, but instead show a steady increase in intracellular calcium that begins at the time of dendritic initiation, when the leading process of the migrating CPN is encountering the MZ. The possible existence of CRN to CPN communication was revealed by the application of veratridine, a sodium channel activator, which has been shown to preferentially stimulate more mature cells in the MZ at an early developmental time. Surprisingly, veratridine application also triggers large calcium transients in CPNs, which can be partially blocked by a cocktail of antagonists that block glutamate and glycine receptor activation. These findings outline a model in which CRN spontaneous activity triggers the release of glutamate and glycine, neurotransmitters that can trigger intracellular calcium elevations in CPNs. These elevations begin as CPNs initiate dendritogenesis and continue as waves in the post-migratory cells. Moreover, we show that the pharmacological blockade of glutamatergic signaling disrupts migration, while forced expression of a bacterial voltage-gated calcium channel (CavMr) in the migrating neurons promotes dendritic growth and migration arrest. The identification of CRN to CPN signaling during early development provides insight into the observation that many autism-linked genes encode synaptic proteins that, paradoxically, are expressed in the developing cortex well before the appearance of synapses and the establishment of functional circuits.

## 1. Introduction

The apical dendrite is initiated by the direct transformation of the leading process of the migrating neuron during the terminal phase of migration [1,2,3]. During this ~2 h period, the dendrite grows ~2.5-fold into the marginal zone (MZ), the cell sparse outer layer of the developing cortex that contains the Cajal-Retzius neurons (CRNs) and their axons [3,4,5]. Most, if not all, developing excitatory Cortical Projection Neurons (CPNs) encounter CRNs at the endpoint of their extended radial migration route from the subventricular zone (SVZ) through the intermediate zone (IZ), subplate (SP), and developing cortical plate (CP) [6]. Thus, CRNs located in the MZ are ideally posed to promote the transition from migration to differentiation status for these immature cortical neurons. CRNs secrete an essential glycoprotein called Reelin [7,8,9] that is required for both normal cellular positioning (layer formation) and normal dendritic initiation and growth [2,3,10]. In the absence of Reelin signaling, neurons can migrate, but will fail to migrate through existing layers of cortical neurons [11], and will later exhibit dysmorphic dendrites [1].

This observation that immature dendrites of excitatory neurons project into a plexus of axons in a highly regulated fashion raises the question of the purpose of this projection. CRNs are spontaneously active [12,13,14]; however, Reelin secretion does not appear to be activity-dependent [15,16,17], and current models suggest Reelin or its proteolytic fragments might diffuse from the MZ [18], implying that close dendrite axon proximity is not required for Reelin signaling itself. While it is appealing to think that this projection anticipates future synapse and circuit formation, it is important to recognize that cortical CRNs are a transient cell class that dies off postnatally [19] and most L6 neurons do not maintain an apical dendrite in the MZ. Thus, this rich interweaving of processes of the CP and CR neurons is transient and likely only maintained for days during mouse cortical development.

Neuronal activity is understood to be essential for the appropriate development of the nervous system [20], and multiple roles have been identified for amino acid neurotransmitters during development. Glutamatergic [21] and GABAergic [22,23] signaling can control neuronal migration and separately dendritic filopodia [24,25,26]. During the embryonic period, these neurotransmitters can elevate intracellular calcium levels depending on the expression of specific classes of receptors [20]. More recently, experimentally enhanced neuronal activation and elevation of intracellular calcium either by sodium channel misexpression [27] or Designer Receptors Activated by Designer Drugs (DREADDs) [28] have been shown to cause ectopic migration arrest and dendritic initiation. However, to our knowledge, it is not known whether intracellular calcium increases occur naturally during dendritic initiation, nor have the potential source of the triggering neurotransmitters been identified.

In this study, we examine the hypothesis that CRNs can communicate to migrating CPNs using amino acid neurotransmitters. Using whole hemisphere explants and genetically encoded calcium indicators, we provide several observations consistent with this hypothesis. First, we demonstrate that migrating CPNs show an increase in intracellular calcium levels as they approach the MZ, during the period of leading process branching. We also show that CRNs are spontaneously active from the earliest stages of cortical layer development when layer 6 is assembling. Moreover, stimulation of CRNs is known to cause the release of glutamate and glycine [29,30,31], and we find that glutamatergic and glycinergic antagonists block stimulated calcium elevations in CPNs. Significantly, the application of these same antagonists as well as tetrodotoxin (TTX) rapidly depresses intracellular calcium levels of CPNs. Lastly, we show that experimental manipulations that alter intracellular calcium levels in migrating neurons disrupt migration and cause excessive dendrite growth.

## 2. Results

### 2.1. Characterization of Intracellular Calcium Signals during Dendritic Initiation and Migration Arrest

**Figure 1:**
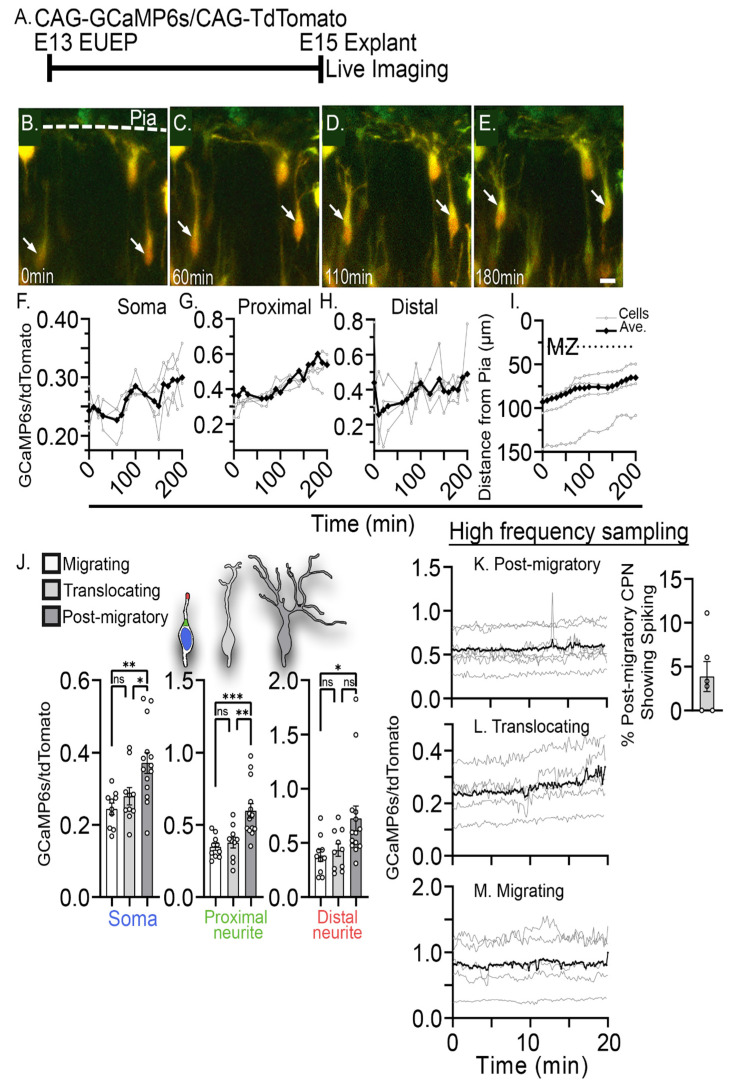
Intracellular calcium signals (GCaMP6s) during migration arrest and dendritic initiation. (**A**) Experimental design: CAG-GCaMP6s and CAG-tdTomato plasmids were co-electroporated into ventricular progenitors of the developing cortex on E13. Whole hemispheres were cultured for 2 days before 2-photon live imaging. (**B**–**E**) Live imaging of dendritic initiation (1 z-stack/10 min) for a 3 h period. Arrows identify CPNs transitioning from migratory to post-migratory stages. (**F**–**H**) Quantified GCaMP6s/tdTomato signals derived from regions of interest (ROI) in the (**F**) soma (blue), (**G**) proximal dendrite (green), and (**H**) distal dendrite (red) as neurons (**I**) translocate and begin dendritogenesis, MZ: Marginal zone (dotted line). (**J**) Comparison of the calcium signal at successive stages of CPN development: migrating (M), translocating (T), and post-migratory (PM). M and T neurons had similar proximal GCaMP6s signals of 0.24 ± 0.05 and 0.28 ± 0.08, respectively. In contrast, PM neurons revealed a significantly higher GCaMP6s signal of 0.37 ± 0.11. PM vs. T (*p* = 0.04), PM vs. M (*p* = 0.003), M vs. T (*p* = 0.74). In the proximal neurite, GCaMP6s was 0.60 ± 0.19 in PM vs. 0.35 ± 0.08 and 0.38 ± 0.12 in M and T neurons, respectively. PM vs. T (*p* = 0.002), PM vs. M (*p* = 0.0006), M vs. T (*p* = 0.96). Finally, in the distal neurite GCaMP6s signal of PM neurons were 0.73 ± 0.42 vs. 0.38 ± 0.17 and 0.43 ± 0.18 in M and T neurons, respectively, with significance of PM vs. T (*p* = 0.08), PM vs. M (*p* = 0.03), and M vs. T (*p* = 0.98). (**K**–**M**) High-frequency sampling (1 z-stack/7 s) in GCaMP6s expressing PM CPNs. Only 3.9 ± 1.7% of PM CPNs showed spiking activity during 20 min of imaging (*n* = 4 explants). (**L**) translocating and (**M**) migrating neurons showed no spiking activity. All comparisons were made using ordinary one-way ANOVA and Šidák’s multiple comparisons. Significance: ns *p* > 0.05, * *p* ≤ 0.05, ** *p* ≤ 0.01, *** *p* ≤ 0.001. Scalebar: 10 μm.

To gain a better understanding of the calcium signal associated with apical dendrite initiation, a construct expressing the genetically encoded calcium indicator GCaMP6s [32] with a Ca^2+^ Kd of 110 nM, was co-electroporated with a construct expressing tdTomato [33] at E13 to sparsely label migrating and differentiating cortical neurons. Whole hemisphere explants were then prepared and allowed to develop for 2 days in vitro [34] and then imaged at E15, as the first groups of electroporated neurons had formed a layer underneath the CRNs and many neurons were still in the process of radial migration (Figure 1A). GCaMP6s (green ) signaling intensity was ratioed against tdTomato (red) signal intensity to control for variations in tissue depth and laser intensity.

We examined the calcium signal of the neurons as they transitioned from radial migration through translocation and early dendritogenesis. We have previously shown that migrating neurons undergo a consistent morphological transformation during dendritic initiation, in which the leading process of the migrating neuron branches and elaborates dendritic filopodia into the MZ during the final two hours of radial migration [3]. Multiphoton imaging of whole hemisphere explants revealed a low GCaMP6s/tdTomato ratio in migrating neurons. However, this ratio slowly increased during the 2 h translocation period (Figure 1B–E). Quantifying the soma, proximal dendrite, and distal dendrite revealed a similar slow increase in the GCaMP6s/tdTomato ratio (Figure 1F–H). This increase was confirmed by comparing the calcium signal of migrating, translocating, and post-migratory neurons at a single time point (Figure 1J). Post-migrating neurons showed the highest calcium signal compared to migrating and translocating neurons. This steady increase during translocation was observed regardless of sampling frequency from 1 z-stack per 10 min to 1 z-stack per 7 s. We did not observe rapid high amplitude signal (signal changes > 1.4 F/F_0_) in the migrating and translocating neurons. However, some spiking was seen in post-migrating neurons (Figure 1K). Only 4% of post-migrating neurons showed any spiking activity within a 20 min imaging period (*n* = 17). This ~2-fold increase in GCaMP6s signal raises the possibility that the rise in intracellular calcium may contribute to dendritic initiation and migration arrest.

**Figure 2:**
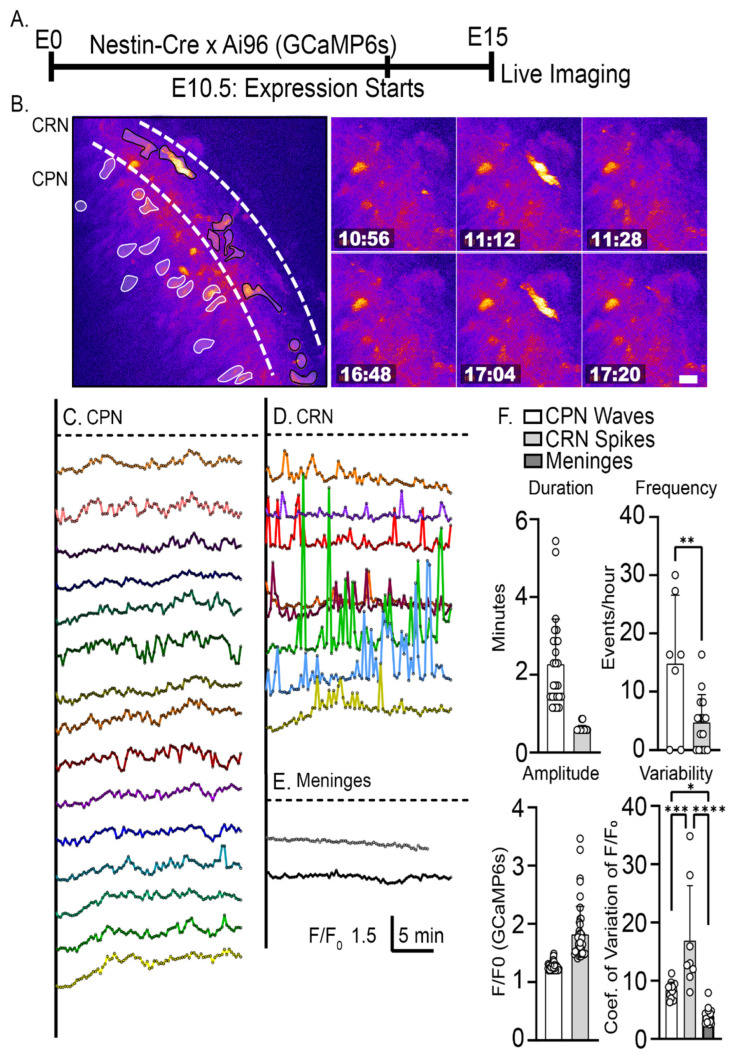
Spontaneous calcium transients during early cortical development. (**A**) Experimental design: embryonic hemispheres from a Nestin-Cre X Ai96 (GCaMP6s) transgenics are imaged using 2-photon microscopy on E15 at one z-stack per 9 s intervals. (**B**) CRN (black outline) and CPN (white outline) are identified in the imaged field by their morphology and respective positions in the MZ and CP. (**C**–**E**) GCaMP6s calcium activity in CPNs, CRNs, and control signal (auto-fluorescence) in the meninges. (**F**) Quantitative characterization of intracellular calcium waves and spikes. CPNs exclusively exhibit calcium waves with an amplitude of 1.26 ± 0.06 F/F_0_ (mean ± s.d.), frequency of 14.8 ± 11.8 waves/h, and duration of 2.27 ± 1.2 min. CRNs exhibit calcium spiking with a mean amplitude of 1.82 ± 0.48 F/F_0_, frequency of 4.73 ± 4.8 spikes/h, and duration of 0.60 ± 0.09 min. CRN F/F_0_ had an average coefficient of variation (CV) of 16.9 ± 9.4 compared to CPN of 8.4 ± 1.4. Autofluorescence in the meninges was measured to assess internal fluorescent fluctuation. The autofluorescence did not exhibit any spiking or wave activity and had an average CV of 4.01 ± 1.4, which is significantly lower than the fluorescence fluctuations observed with CPN signal. Significance: * *p* ≤ 0.05, ** *p* ≤ 0.01, *** *p* ≤ 0.001, **** *p* ≤ 0.0001. Unpaired two-tailed *t*-tests were used for comparing duration, frequency, amplitude. Coefficient of variation comparisons were made using ordinary one-way ANOVA and Šidák’s multiple comparisons n = 2 explants, and at least seven neurons from each class. Scale bar: 20 μm.

Prior studies have revealed that CRNs in the superficial layer of the developing cortex are spontaneously active, showing intracellular calcium transients that last between 10–100 s [35,36]. At early time points in development, the activity is uncorrelated among CRNs, but as development proceeds, correlated activity increases, indicating the formation of a network among the CRNs. However, it is unclear whether CRN activity, at any time point, correlates with the activity of the CPNs in the underlying cortical plate. To examine the possibility of CRN to CPN communication, we crossed the Ai96 line, which conditionally expresses the calcium indicator, GCaMP6s [37,38], with a Nestin-Cre line that drives recombination in the mouse CNS starting at embryonic day 10.5 (E10.5) [39]. On E15, whole hemisphere explants were prepared (Figure 2A), a procedure that does not involve tissue slicing and keeps the meninges and underlying neural tissue intact [34]. This approach provided GCaMP6s expression in both the CRNs and the underlying CPNs.

Multiphoton imaging of Nestin-Cre: Ai96 hemispheres was performed through the intact meninges in a region of the dorsal medial cortex that includes primary and secondary motor cortex areas. In this configuration, the sparse, large, and horizontally oriented CRNs are at the top of the imaged field, and the densely packed, developing CPNs are vertically oriented below (Figure 2B). At this time in development, the CPNs in the imaged field correspond to presumptive deep-layer neurons, primarily L6 and L5 excitatory neurons [40]. z-stacks were collected at 9-s intervals for periods of up to 30 min. Neuronal calcium signals can be broadly characterized as spikes and waves by amplitude and kinetics [41]. In the present study, spikes were defined as changes in the F/F_0_ exceeding 1.4 and having a duration of less than 1 min, while waves were defined as exceeding 1.2 F/F_0_ and being longer than 1 min. CPNs exhibit calcium waves with an amplitude of 1.26 ± 0.06 F/F_0_ and a frequency of 14.8 ± 11.8 waves/h (mean ± s.d.), and a duration of 2.27 ± 1.2 min (Figure 2C,F). CRNs exhibit a form of calcium spiking with a mean amplitude of 1.82 ± 0.48 F/F_0_, frequency of 4.7 ± 4.8 spikes/h, and duration of 0.60 ± 0.09 min (Figure 2D,F). The 9-s z-stack intervals slightly under sample the shorter duration spike transients and make it likely the mean spike amplitude and the number of spikes were underestimated. As an internal fluorescent fluctuation control, the meninges fluorescence signal was also measured (Figure 2E,F). The meninges did not exhibit any spiking or wave activity as defined in this study. Although the spike amplitude and duration are approximately consistent with prior studies, the observed spike rate is higher than in previous reports. This difference may reflect the early time point (E15) examined in our study [12,35], the fact that the meninges are left intact in our whole hemisphere preparation [12], the use of isoflurane anesthesia for in utero imaging [36], or the presence of excitatory amino acids in the DMEM-F12 culture media employed in our study. There was no obvious temporally correlated spiking among the CRNs, which typically corresponded to ~15 CRNs per imaged field, although this issue was not investigated in depth. In contrast, the underlying CPNs showed very infrequent spiking behavior and instead exhibited smaller amplitude wave-like calcium transients consistent with previous descriptions of elicited transients in CPNs [42]. While these data did not identify correlated spiking between CRN and CPN during early cortical development, they leave open the possibility of correlated CRN spike to CPN wave activity.

**Figure 3:**
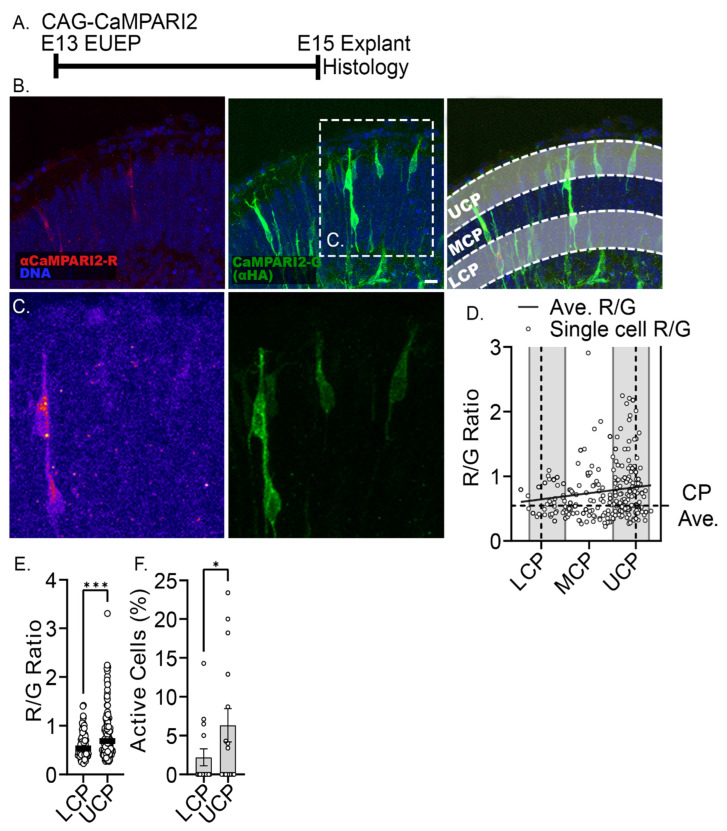
Fixed calcium signal confirms intracellular calcium signals (CaMPARI2 R/G ratio) are higher in neurons located in the upper cortical plate (UCP) compared to neurons in the lower cortical plate (LCP). Middle cortical plate (MCP) quantification is not shown. (**A**) Experimental design: CAG-CaMPARI2 plasmid was electroporated into the developing cortex on E13. Whole hemispheres were cultured for 2 days before photoconversion and paraformaldehyde fixation of the CaMPARI2 signal. (**B**) Calcium-bound CaMPARI2 is shown in red. Total CaMPARI2 is revealed by antibody detection of the HA tag on the CaMPARI2 protein (green). (**C**) Inset shows a higher magnification view of neurons in the UCP and LCP. The red channel is shown using the Fire lookup table to emphasize intracellular calcium differences. (**D**) Quantification of R/G ratio across the cortical plate (CP). The horizontal dashed line is the average of the CP R/G ratios. The solid line shows a simple linear regression line of the R/G ratios across the CP and reveals an elevated R/G ratio in the UCP compared to LCP. (**E**) R/G ratios from neurons in the UCP (0.82 ± 0.50, mean ± s.d.) vs. in the neurons in the LCP (0.61 ± 0.27), *p* = 0.0002. Black bars represent the mean. (**F**) Percent of active cells (R/G > 0.45) in the UCP (6.33 ± 8.3) compared to the LCP (2.19 ± 4.2), *p* = 0.049. Unpaired one-tailed *t*-tests were used for comparing the R/G ratio and % active cells. Significance: * *p* ≤ 0.05, *** *p* ≤ 0.001. n = 5 explants. Scale bar: 10 μm.

As the intracellular calcium change that correlates with the period of dendritic initiation is relatively slow and steadily increasing, we initiated studies using the fixable calcium indicator CaMPARI2 F391W (Ca^2+^ Kd = 110 nM), which reports calcium levels at the time of photoconversion and fixation [43]. Calcium-bound CaMPARI2 fluoresces red after photoconversion, while in our experiments, total CaMPARI2 is detected by anti-HA-tag immunofluorescence (green). Thus, the CaMPARI2 Red/Green (R/G) ratio reflects intracellular calcium levels at the time of fixation. In this case, a construct expressing CaMPARI2 F391W was ex utero electroporated, and explants were prepared at E13. After 2 DIV, the explants were photoconverted and fixed for subsequent sectioning and analyses (Figure 3A). Consistent with our live imaging data, neurons with a migration morphology in the upper third of the cortical plate exhibited 1.34-fold higher CaMPARI2 signals than neurons at the lower third of the cortical plate (*p* < 0.001), and neurons with translocation morphology showed increasing CaMPARI2 signals as they neared the CRNs (Figure 3B–E). Moreover, the fraction of high R/G signal (R/G > 0.45) neurons was greater in the upper cortical plate (Figure 3F). Thus, the live and fixed approaches to calcium imaging confirm that intracellular calcium levels increase in CPNs as they approach the spontaneously active CRNs.

### 2.2. Veratridine Stimulation of CRNs Induces Rapid Calcium Transients in CPNs: Partial Block by Glutamatergic and Glycinergic Antagonists

To better understand the potential communication mechanism between CRNs and CPNs, including those CPNs undergoing dendritic initiation, we chemically stimulated explants with veratridine [44]. Veratridine (VRT) is a steroidal alkaloid that increases neuronal excitability by preventing the inactivation of voltage-gated sodium channels (VGSCs). Although this agonist is not selective for CRNs, at E15, CRNs express VGSCs and are excitable [45]. In contrast, CPNs at E14–E16 are inexcitable and show minimal voltage-dependent sodium currents compared to later development [46]. Importantly, VRT application has also been shown to trigger the release of glutamate and glycine (but not GABA), leading to calcium elevations in E13 neurons of the developing preplate (PP), including CRNs [29]. Thus, VRT stimulation likely triggers the release of neurotransmitters from CRNs that might elicit intracellular calcium elevations in CPNs.

**Figure 4:**
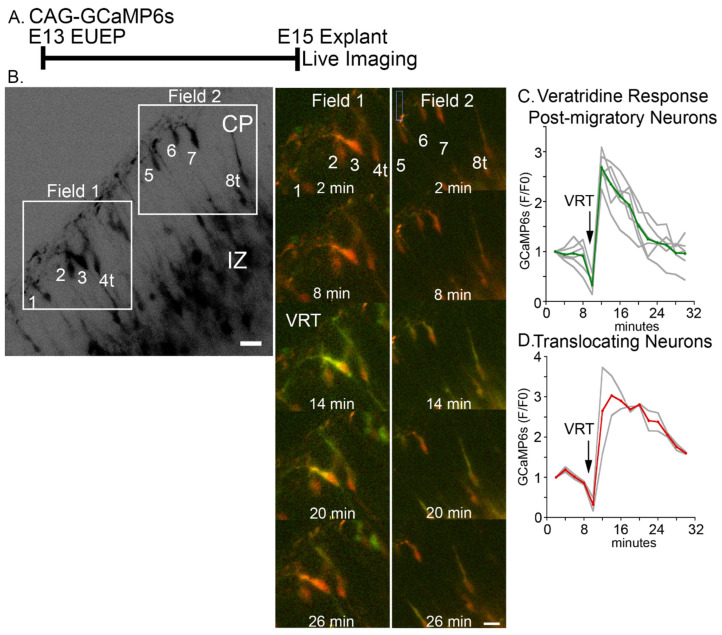
Veratridine (VRT) stimulation targeting CRNs induces a rapid calcium (GCaMP6s) signal transient in CPNs. (**A**) Experimental design: CAG-GCaMP6s plasmid was electroporated into the developing cortex on E13. Whole hemisphere explants were cultured for 2 days before 2-photon live imaging. (**B**) Example field of neurons imaged during live GCaMP6s imaging (CP: Cortical plate, IZ: Intermediate zone). Cells were analyzed in two areas (Field 1 and Field 2) and revealed a calcium transient approximately 2 min after bath application of VRT at 12 min. (**C**,**D**) Quantification of the VRT response in (**C**) post-migratory and (**D**) translocating neurons revealed a 2–3-fold increase in calcium signal (F/F_0_) from both populations. Gray lines are individual traces, red and green lines are the average. Scale bar: 15 μm.

**Figure 5:**
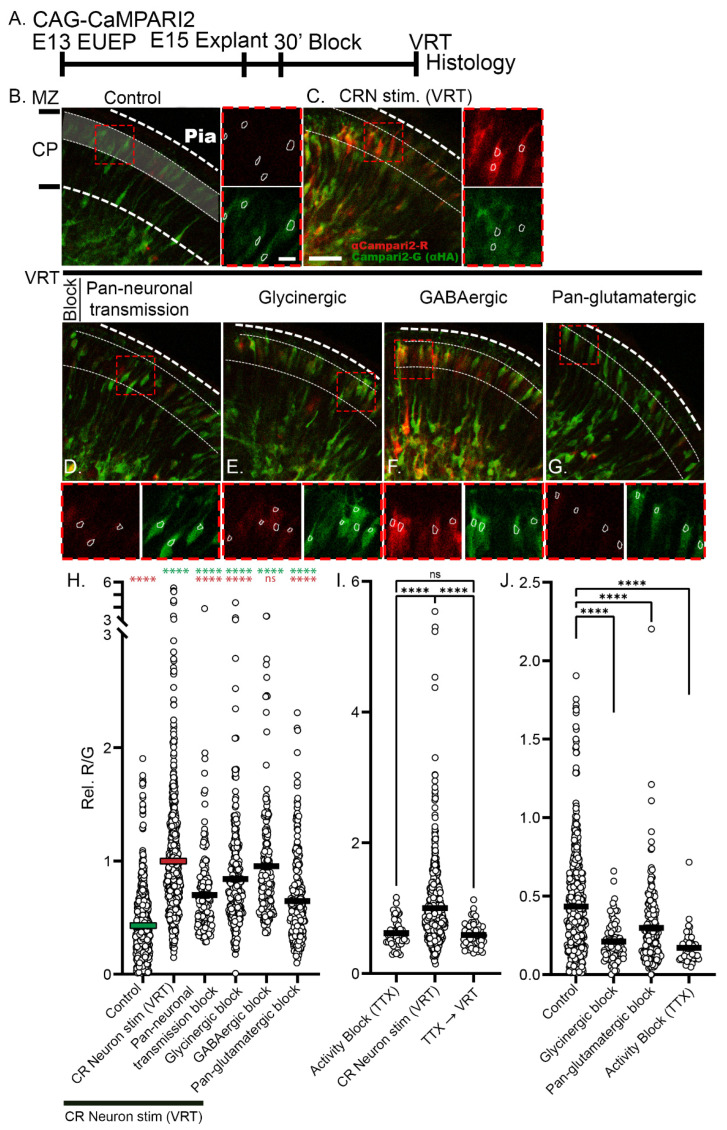
Veratridine (VRT) stimulation targeting CRNs elevated the intracellular calcium signal (CaMPARI2 R/G) in CPNs, and glutamatergic and glycinergic antagonists partially block the response. (**A**) Experimental design: CAG-CaMPARI2 plasmid was electroporated on E13 and whole hemisphere explants were cultured for 2 days prior to pharmacological challenge on E15. (**B**,**C**) VRT caused an increase in the R/G ratio in post-migratory CPN in the UCP (MZ: Marginal zone, CP: Cortical plate). (**D**–**G**) CaMPARI2-expressing explants were incubated for 30 min with antagonists before VRT stimulation. (**H**) The highlighted region in (**B**) shows the area of neuronal quantification in the UCP. Bars indicate the mean value. VRT caused a 2.3-fold increase in R/G ratio compared to control (1.0 ± 0.02 vs. 0.43 ± 0.013, respectively, *p* < 0.0001). Pan-neuronal block reduced the VRT to 1.63-fold of control (0.70 ± 0.03), *p* < 0.0001). Glycinergic block reduced the VRT 1.95-fold of control (0.84 ± 0.03, *p* < 0.0001). GABAergic block did not significantly affect the VRT response (0.95 ± 0.03, *p* = 0.87). Pan-glutamatergic block reduced the VRT response to 1.51-fold over control (0.65 ± 0.02, *p <* 0.0001). (**I**) TTX (tetrodotoxin) pretreatment reduced VRT response to 1.44-fold over control (0.63 ± 0.03 vs. 1.0 ± 0.02, *p* < 0.0001) (**J**) In the absence of VRT stimulation, 30 min pretreatment with a glycinergic antagonist (0.21 ± 0.01, *p*<0.0001), pan-glutamatergic antagonists (0.3 ± 0.02, *p* < 0.0001), or TTX (0.17 ± 0.01, *p* < 0.0001) reduced calcium (CaMPARI2 ratio) compared to control (0.43 ± 0.013), indicating baseline intracellular calcium levels are maintained by ongoing NT signaling. Comparisons were made using ordinary one-way ANOVA and Šidák’s multiple comparisons. Data from a minimum of five explants in each condition. Significance: ns *p* > 0.05, **** *p* ≤ 0.0001 (Labels in red are a comparison to VRT, labels in green are a comparison to Control). Scale bar: 30 μm. Outset scalebar: 10 μm.

To measure the spatial and temporal response to veratridine, we first performed E13 GCaMP6s EUEPs and prepared explants for E15 live imaging (Figure 4A). VRT treatment caused a rapid rise in calcium signal in both translocating and post-migratory CPNs (Figure 4B–D). The response occurred within 2 min of VRT application to the imaging bath and exceeded F/F_0_ values of 2. The calcium signal was detected throughout the dendritic arbor and soma in post-migratory neurons and migratory CPNs underneath the MZ. We next tested VRT application to E15 whole hemisphere explants electroporated with CaMPARI2 (Figure 5A). VRT increased the intracellular calcium signal in both post-migratory CPNs directly under the CRN and migratory CPNs approaching the CRN (Figure 5B,C). The R/G ratio of VRT-treated cells was 2.3-fold higher than control (1.0 ± 0.02 vs. control 0.43 ± 0.013, mean ± s.e.m., *p* < 0.0001) (Figure 5B,C,H). In addition, we observed significant calcium signal increases in multipolar neurons (MPNs) in the IZ and in the areas underlying the SP (Figure 5C).

In order to ascertain if the calcium increases in CPNs were a result of neurotransmitter (NT) release rather than direct VRT stimulation of CPNs and MPNs, we conducted a preincubation of the explant with a mixture of neurotransmission inhibitors (as described in the Methods section) before applying VRT. The pan-neuronal transmission cocktail targeted glutamatergic, GABAergic, and glycinergic receptors known to be expressed at the mRNA level by these neurons at this developmental time point [47]. While VRT produced 2.3-fold increase in CaMPARI2 signal (1.0 ± 0.02), compared to control (0.43 ± 0.013), preincubation with the pan-neuronal blockers reduced the VRT signal to 1.6-fold (0.70 ± 0.03), a value approximately midway between VRT and control values (*p* < 0.0001). Applying the glycinergic blocker strychnine also partially blocked the VRT response, but to a much lesser extent than the pan-NT blocker (0.84 ± 0.03, *p* < 0.0001). GABA has been shown to depolarize some classes of embryonic neurons; however, the GABAergic blocker (bicuculline) did not affect the VRT response (0.95 ± 0.03, *p* = 0.87). Significantly, the pan-glutamatergic block was as effective as the pan-neuronal block in suppressing the VRT response, reducing the VRT stimulation to 1.5-fold over control (0.65 ± 0.02, *p* < 0.0001) (Figure 5D–H). Thus, preincubation with the inhibitors partially blocked the response to VRT, suggesting that VRT triggers the release of NTs, including glutamate and glycine, that contribute to calcium elevations in the CPNs and MPNs.

### 2.3. Reduction of Baseline Intracellular Calcium Signal by Glutamatergic and Activity Blockade

**Figure 6:**
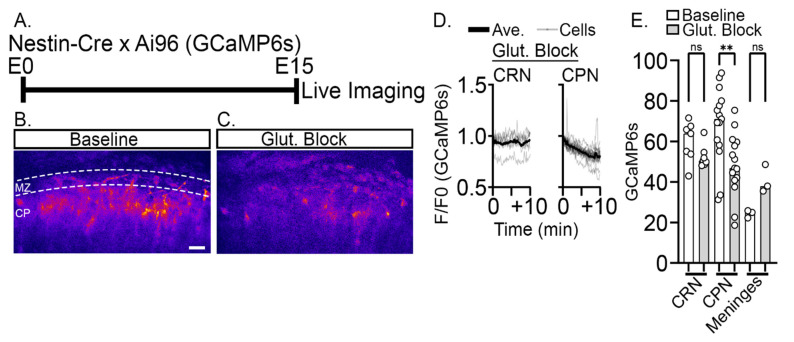
Glutamatergic antagonists rapidly lower baseline intracellular calcium (GCaMP6s) signal in CPN. (**A**) Experimental design: Nestin-Cre X Ai96 (GCaMP6s) hemispheres were cultured on E15, and antagonists were applied at Time = 0 while imaging. (**B**) Baseline intracellular calcium (GCaMP6s) levels and (**C**) calcium levels 10 min after glutamatergic antagonist application. (Shown in the Fire lookup table, MZ: Marginal zone, CP: Cortical plate) (**D**) Traces of somal signal in both CRNs and CPNs during imaging. (**E**) Glutamatergic antagonists did not alter GCaMP6s signal in CRNs (52.6 ± 2.1, mean ± s.e.m.) vs. baseline (60.1 ± 3.8, *p* = 0.70). Glutamatergic blockade did disrupt GCaMP6s signal in the CPNs (48.4 ± 4.0) relative to baseline (68.5 ± 4.8, *p* = 0.001). There was no significant difference between the treatment groups in the meninges (baseline 24.3 ± 1.1 vs. block 40.7 ± 4.0, *p* = 0.42). Comparisons were made using ordinary one-way ANOVA and Šidák’s multiple comparisons. n = 2 explant, at least 7 neurons of each class. Significance: ns *p* > 0.05, ** *p* ≤ 0.01. Scale bar: 20 μm.

To determine if VRT was specifically activating voltage-dependent sodium channels, we preincubated the explants with tetrodotoxin (TTX), a highly selective sodium channel blocker [48]. TTX preincubation completely blocked VRT-induced calcium increases, suggesting that VRT response is not generated by off-target effects (Figure 5I). TTX alone-treated (0.62 ± 0.03) were not different than TTX plus VRT-treated (0.63 ± 0.03), but both TTX groups were significantly lower than VRT stimulation alone (1.0 ± 0.02, *p* < 0.0001). We next asked if TTX, glycinergic, or pan-glutamatergic blocks alone would lower baseline CaMPARI2 signals, which would be expected if ongoing neurotransmitter signaling is required to maintain intracellular calcium levels observed in CPN. In comparison to untreated control R/G value (0.43 ± 0.013), 30 min TTX treatment reduced the signal to 39% of control (0.17 ± 0.01), 30 min treatment with glycinergic blocker reduced the signal to 48% of control (0.21 ± 0.01), and 30 min pan-glutamatergic block reduced the signal to 69% of control (0.30 ± 0.02) (Figure 5J). Multiphoton imaging was then conducted to examine the time course of the response in Nestin-Cre: Ai96 explants on E15 after applying pan-glutamatergic inhibitors (Figure 6A). Baseline GCaMP6s signal began to decline immediately in CPNs but not CRNs after bath application of the blockers, and CPN calcium signal stabilized at a lower level by approximately 10 min (Figure 6B–E). Collectively, the highly significant reduction of baseline intracellular calcium levels by these blockers indicates that ongoing neurotransmitter signaling is required to maintain intracellular calcium levels in CPNs. This is likely mediated by a combination of neurotransmitters, including glutamate and glycine released by CR neurons.

### 2.4. 24-h Pan-Glutamatergic and Activity Blockade Decreased Neuronal Migration into the CP

**Figure 7:**
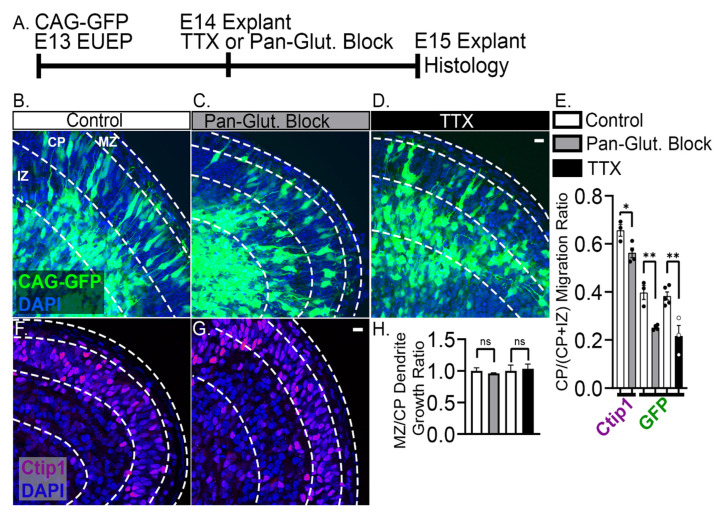
Pan-glutamatergic blockade and activity blockade for 24 h reduces the number of neurons migrating into the CP. (**A**) Experimental design: CAG-GFP plasmid was electroporated into the developing cortex on E13. Whole hemisphere explants were cultured for 1 day before being incubated with either pan-glutamatergic blockers or activity or TTX for an additional 24 h. (**B**,**C**,**E**) Pan-glutamatergic blockade reduces the percent of GFP+ neurons in the CP (38% reduction, *p* = 0.001) (MZ: Marginal zone, CP: Cortical plate, IZ: Intermediate zone). (**D**,**E**) Activity blockade by TTX also caused a 42% reduction of GFP+ neurons in the CP (*p* = 0.003). (**E**–**G**) Similarly, a 15% reduction of CTIP1 immunopositive neurons in the CP was found with pan-glutamatergic blockade (*p* = 0.014). (**H**) No differences were seen in dendritic growth into the MZ in either pan-glutamatergic blockade or activity blockade groups. Unpaired one-tailed *t*-tests were used to compare the MZ/CP dendrite growth ratios and the CP/(CP + IZ) migration ratios. Data from a minimum of three explants in each condition. Significance: ns *p* > 0.05, * *p* ≤ 0.05, ** *p* ≤ 0.01. Scale bar: 10 μm.

To assess the phenotypic consequences of glutamatergic and activity blockade, we performed EUEPs with CAG-GFP to label developing neurons. Then we exposed whole hemisphere explants to pan-glutamatergic blockers 24 h before fixation and analyses on E15 (Figure 7A). Chronic exposure to the blockers altered the distribution of labeled cells across the cerebral wall and reduced the fraction of GFP+ neurons found in the CP compared to the IZ (Figure 7B–E) [49]. In control explants, 40% of GFP+ neurons were found in the CP, whereas TTX caused a reduction to 22%, and pan-glutamatergic blockade caused a decrease to 25%. The result was confirmed by examining the immuno-distribution of Ctip1, a transcription factor that identifies early born neurons. Pan-glutamatergic blockers reduced the percentage of Ctip1+ neurons in the CP to 85% of control values (Figure 7E–G), as would be expected, since Ctip1+ represents the distribution of all CPNs rather than focusing on the migratory and immediate post-migratory populations identified by GFP+. These results suggest that migration is delayed or arrested in the presence of blockers. This finding is consistent with prior studies suggesting glutamatergic signaling modulates migration rate in the cerebral cortex [21].

Closer inspection of the migrating neurons did not reveal any obvious morphological distinctions between blocked and control conditions. To determine the consequence of glutamatergic blockade on dendritic growth in the MZ, we measured the above threshold GFP fluorescence pixel area in the MZ that represents the dendritic projection, and divided that value by the above threshold GFP fluorescence pixel area in the underlying CP, a proxy measurement for the number of neuronal soma [2,50]. However, quantifying dendrite projection into the MZ did not reveal a reduction with either blocker (Figure 7H).

### 2.5. Misexpression of Voltage-Dependent Sodium and Calcium Channels Reduced Migration into the CP and Enhances Dendritic Growth in the MZ

**Figure 8:**
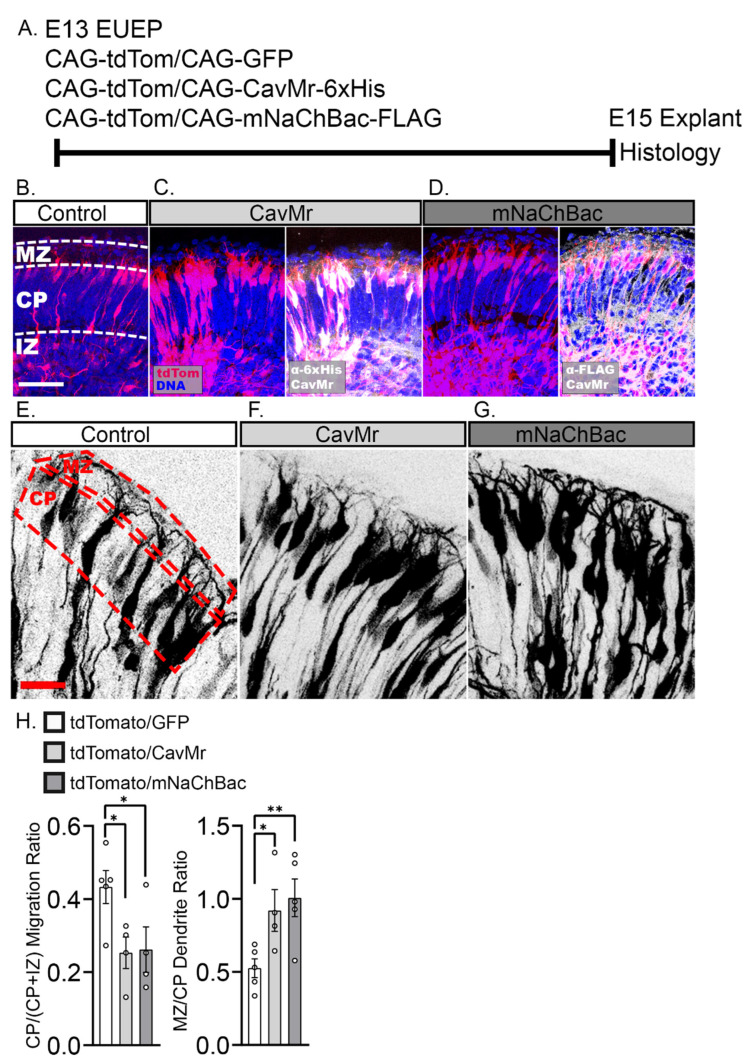
Misexpression of a voltage-dependent sodium channel (bacterial mNaChBac) or a voltage-dependent calcium channel (bacterial CavMr) inhibited migration into the CP and increased dendritic growth into MZ. (**A**) Experimental design: Control CAG-GFP or ion channel expressing plasmids were co-electroporated with CAG-tdTomato plasmid on E13. Whole hemispheres were cultured for 2 days before being processed for histology. (**B**) Control section compared with (MZ: Marginal zone, CP: Cortical plate, IZ: Intermediate zone) (**C**) CavMr and (**D**) mNaChBac misexpression sections and immunostained for 6×His and FLAG tags, respectively. Greater fractions of neurons were found below the CP in the ion channel misexpressing explants compared to control (**E**–**G**). Higher magnification image of the upper CP/MZ showing increased dendritic growth into the MZ in explants expressing CavMr and mNaChBac. (**H**) CavMr misexpression caused a 42% decrease in the CP/(CP + IZ) migration ratio compared to control (*p* = 0.029). mNaChBac misexpression caused a 40% decrease in CP migration ratio compared to control (*p* = 0.035). However, compared to control (0.53 ± 0.06), dendritic projection in the MZ was increased by 74% after CavMr misexpression (0.92 ± 0.14, *p* = 0.034) and increased 91% by mNaChBac (1.01 ± 0.13, *p* = 0.009). Comparisons were made using ordinary one-way ANOVA and Šidák’s multiple comparisons. Data from a minimum of four explants in each condition. Significance: * *p* ≤ 0.05, ** *p* ≤ 0.01. Scale bars: (**B**–**D**), 50 μm (**E**–**G**), 20 μm.

Prior studies showed that enhancing intracellular calcium transients in migrating cortical neurons by forced expression of a voltage-dependent sodium channel [27] or by activating DREADD (Designer Receptor Activated by Designer Drug) [28] led to migration arrest and ectopic dendritic initiation. These results raise the possibility that intracellular calcium elevation naturally triggers migration arrest and dendritogenesis at the end of the migration period. To directly test whether calcium elevations in migrating neurons are sufficient to trigger migration arrest and ectopic dendritogenesis, we misexpressed the prokaryotic voltage-dependent calcium channel CavMr [51] by EUEP at E13. As a positive control, we separately expressed the bacterial sodium channel mNaChBac (Figure 8D), which was shown to drive migration arrest and ectopic dendritogenesis [27]. We then examined the distribution of neurons at E15 (Figure 8A–C). In both cases, the forced expression of these channels altered neuronal distribution and reduced the CP/(CP + IZ) fraction, indicative of a migration delay or arrest (Figure 8H). In the deep areas, more neurons appeared to have elaborated neurites; however, it was impossible to determine whether the neurons had elaborated ectopic dendrites, given the high density of labeled cells. We measured the dendritic projection ratio (as above) to assess the consequence of ion channel expression on dendritic growth. Consistent with this interpretation, we found that chronic ion channel expression increased the dendrite projection ratio in the MZ by approximately >1.5-fold for both channels (Figure 8E–H). The ion channel findings suggest that intracellular calcium levels control migration speed and dendritic growth.

### 2.6. Ethanol Exposure Did Not Alter the Baseline CaMPARI2 Signal or Disrupt the Response to Veratridine

**Figure 9:**
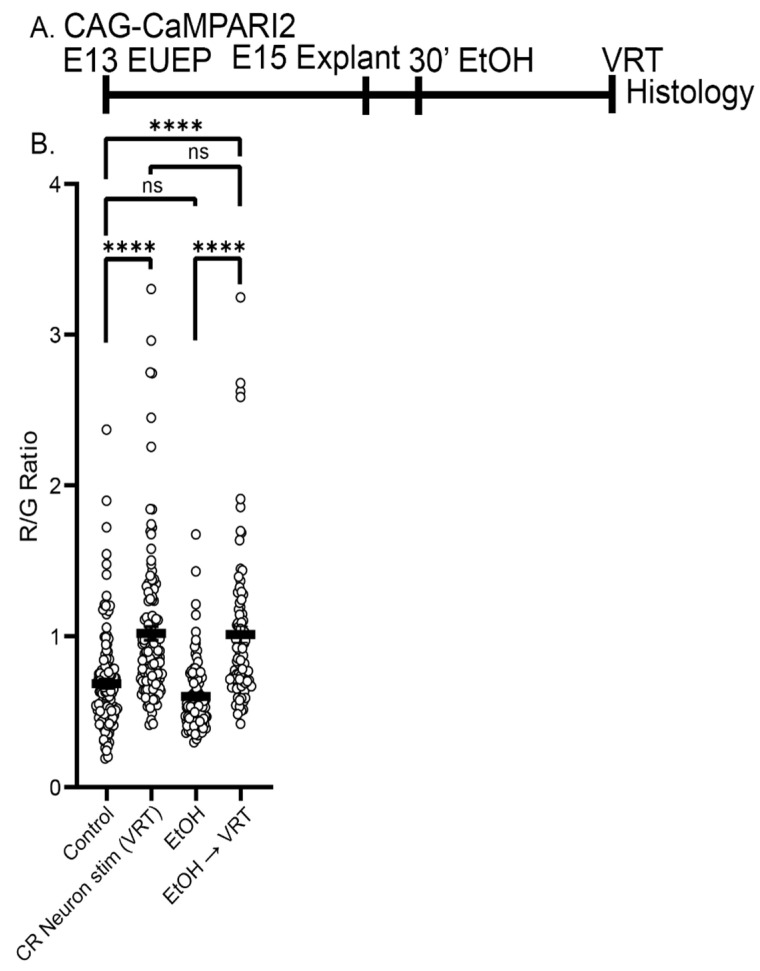
Ethanol exposure does not acutely alter baseline CaMPARI2 signal or disrupt the VRT response. (**A**) Experimental design: CAG-CaMPARI2 plasmid was electroporated into ventricular progenitors of the developing cortex on E13. Whole hemispheres were cultured for 2 days before ethanol (EtOH) treatment and processing for histology. (**B**) There was no significant difference in CaMPARI2 signal between EtOH-treated (400 mg/dL, 30 min) explants and control (0.69 ± 0.03 vs. 0.60 ± 0.02, *p* = 0.64). EtOH pretreatment also failed to block the VRT response (1.02 ± 0.05 vs. 1.01 ± 0.05, *p* > 0.99). Black bar represents the mean. Comparisons were made using ordinary one-way ANOVA and Šidák’s multiple comparisons. Significance: ns *p* > 0.05, **** *p* ≤ 0.0001. Data from a minimum of three explants in each condition.

Cortical neuron development is disrupted by ethanol exposure [52,53,54], in part due to its ability to inhibit glutamatergic neurotransmission [55,56,57]. Moreover, CRN physiology is sensitive to ethanol exposure later in development [58]. In parallel, we have found that acute exposure to ethanol rapidly disrupts dendritic outgrowth [59,60]. To determine if acute ethanol exposure disrupts CRN to CPN signaling, CaMPARI2-expressing explants were preincubated for 30 min with ethanol (400 mg/dL) or control media before VRT stimulation on E15 (Figure 9A). However, no difference in the amplitude of the VRT response was observed between control 1.02 ± 0.05 and ethanol 1.01 ± 0.05 pretreated cells (*p* > 0.99) (Figure 9B). Moreover, there was no significant difference in baseline R/G levels between control (0.69 ± 0.03) and ethanol-treated (0.60 ± 0.02) cells (*p* = 0.64). Although preliminary, this finding suggests that acute disruption of CRN to CPN signaling is not the primary mode of action for this neurodevelopmental toxin.

## 3. Discussion

The initial step in dendritic development is the establishment of cellular polarization [61]. In the developing rodent cortex, this polarization occurs underneath the subplate as multipolar neurons [62] initiate axonal development and attach to the radial glial process. Several cues have been identified that contribute to this polarization, including TgfB [63], radial glial contact [64], and glutamatergic signaling [49]. The next step in cortical migration is canonical radial glial-guided migration, in which CPNs migrate along radial glial processes [65] in a saltatory fashion [66]. During this period, the prospective dendrite is called the leading process and remains simplified and attached to the radial glial fiber. Migrating neurons can show calcium elevations that, in some cells, modulate migration rate [67] and that increase in frequency and amplitude in post-migratory neurons [27,28]. The post-migratory calcium signals likely regulate both neurite outgrowth [26] and patterns of gene expression [68,69,70].

**Figure 10:**
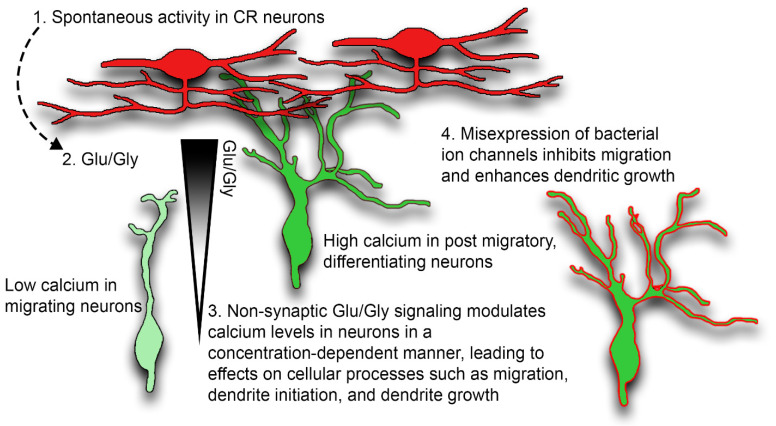
Model of Cajal-Retzius neuron to Cortical Projection Neuron signaling. Intrinsic properties of CRNs generate (1) spontaneous activity in CRNs. Spontaneous activity in the Cajal-Retzius neurons leads to (2) glutamate and glycine secretion. (3) Secreted glutamate and glycine cause additional Cajal-Retzius neuron activity and intracellular calcium elevations, and dendritic growth in Cortical Projection Neurons. (4) Misexpression of bacterial ion channels (CavMr and mNaChBac) prematurely enhances calcium signaling, causing dendritic elaboration and migration arrest.

Our study investigated calcium signaling during dendrite initiation, the period between migration and post-migration (Figure 10). At the end of radial migration, the leading process transforms into a branched dendrite that extends into the MZ [3]. The soma translocates and then arrests underneath the first stable branch point of the nascent dendritic arbor. In prior imaging studies and this study, the elaboration of the branched leading process always preceded migration arrest, suggesting dendritic elaboration may serve as a stop signal for radial migration by preventing further nucleokinesis [2,3]. The combined dendritic deployment and migration arrest is a critical period in the development of a cortical neuron, as it coincides with the expression of hundreds of genes, many of them disease-linked [47,71,72]. While several cues have been identified that are required for apical dendrite development [73,74], the natural trigger for dendritic initiation and migration arrest is unclear.

In developing neurons, intracellular calcium signals in the soma, dendrites, and axons can show independent subthreshold activity reflecting both compartmentalization and diffusion limitation and buffering in the cytoplasm of the neurites. In this study, intracellular calcium levels gradually rise in both the dendrite and soma as migrating CPNs approach CRNs (Figure 10). Consistent with prior studies that enhanced activity in migrating neurons [27,28], we demonstrate that migration arrest can be obtained by forced expression of a voltage-gated calcium channel (Figure 10). Using VRT stimulation, we identify glutamate and glycine as critical amino acid neurotransmitters mediating CRN to CPN signaling (Figure 10), and we show that glutamatergic antagonists cause a rapid drop of intracellular calcium levels in CPNs. Lastly, we and others have found that glutamatergic blockade disrupts migration and dendritogenesis, underscoring glutamate’s importance to these events.

The calcium set point hypothesis proposes that intermediate levels of intracellular calcium support neurite outgrowth, while low levels or high levels of intracellular calcium inhibit growth [75]. While calcium signaling is active during migration, it is still being determined whether the same set point rules govern the process of radial migration. For example, migrating cerebellar granule cells express N-type calcium channels and NMDA receptors [76,77] that contribute to calcium transients that modulate migration [67]. While migrating cortical neurons in acute slice preparations also exhibit calcium elevations, these elevations are small (less than 1.1 F/F_0_) and infrequent (~1/10 min) [27], and these elevations increase in elevation and frequency in post-migratory neurons. In contrast to previous findings, glutamatergic blockade did not alter dendritic growth into the MZ; however, this may be due to the fact that glutamatergic blockade also disrupted migration into the cortical plate, thus eliminating or reducing the number of cells undergoing dendritic initiation during the period of agonist application. However, we confirmed that forced mNaChBac expression drives migration arrest [27] and found that forced expression of CavMr, a voltage-dependent calcium channel, also causes premature migration arrest. Additional evidence for calcium signaling driving migration arrest was obtained using Designer Receptors Activated by Designer Drugs (DREADDs) [78]. Forced expression of the hM3Dq-activating DREADD, which drives IP3-mediated intracellular calcium elevations, caused premature migration arrest and ectopic dendritogenesis [28]. Prior work and our findings suggest that very low levels of intracellular calcium support migration, while higher levels promote dendritic deployment and migration arrest (Figure 10). Critically, our study shows that calcium levels naturally rise during dendritic deployment and migration arrest, and thus, may indeed be triggering dendritic initiation, as would be predicted from these prior studies [28].

It is unclear from existing studies whether external cues trigger the calcium increase, or it is intrinsic, driven by a completely cell-autonomous program. In this work, we present evidence that CRNs are a likely source of the NTs driving dendritic elaboration and migration arrest. The spontaneous activity of CRNs is well established [12]; however, the functional purpose of this activity during early development is unclear. Reelin secretion does not appear to be activity-dependent [15,16,17]; thus, the spontaneous activity observed with CRNs may have other functional importance. During early cortical development, CRNs secrete the neurotransmitters glutamate and glycine, but not GABA, in response to VRT [29]. Although subplate neurons also likely secrete glutamate [49], the proximity of dendrites and axons, as well as the likely activity-dependent secretion of glutamate and glycine, highlight CRNs as the most likely source of the glutamate that is driving calcium elevations during dendritic elaboration and migration arrest.

Glutamate activation of NMDAR is allosterically increased by glycine [79] and NMDAR-mediated glutamate responses have been proposed to control cortical migration, with NMDA antagonist blocking migration in slice culture [21,80] and NMDAR activation causing migratory arrest [81]. Sustained treatment with the NMDAR antagonist MK801 also produces cortical lamination defects in vivo as well [82]. In contrast, genetic studies using global and conditional knockouts of NR1, an essential subunit of the NMDA receptor, did not show disruptions of cortical lamination [83,84,85]. These genetic results suggest that NMDAR deficiency has no impact on cortical migration. Although the reconciliation of these findings will require further work, we have found that both glutamatergic and glycinergic neurotransmitter signaling systems may be involved in establishing cortical neuron calcium levels, suggesting some form of redundancy may be involved.

Moreover, blockade of both glutamatergic and glycinergic signaling did not completely block CRN to CPN signaling, suggesting additional signals contribute to CPN intracellular calcium. Consistent with this multiple signal model, CPNs express mRNAs for other neurotransmitter receptors, including cholinergic receptors, in addition to expressing subunits of NMDA, AMPA, kainate, and metabotropic glutamate receptors, as well as glycine and GABA receptors [28,47]. While glycine is a co-agonist for NMDA receptors [79], like GABA, it can also be depolarizing in the embryonic period after binding glycine receptors [86]. Moreover, applying the glycinergic antagonist strychnine lowered baseline intracellular calcium levels in CPNs, and strychnine is highly selective for the glycine receptors over NMDA receptors. Thus, the observed effects of strychnine are likely due to direct interactions with glycine receptors expressed by CRNs and CPNs [87] rather than NMDA receptors. It is also important to note that CPNs do not show spontaneous action potentials during early development, and only about 1/3 of neurons show an active response to depolarization at this time [46]. This lack of activity in CPNs suggests that the observed calcium fluctuations are primarily due to ligand-gated receptor activity, possibly amplified by release from internal calcium stores.

Glutamate is a critical amino acid for developing neurons, contributing to cell proliferation, cell migration, cell differentiation, and cell survival in the developing cortex [88]. Separating the multiple functional roles of glutamatergic signaling during cortical development is challenging. In this study, the media employed is DMEM-F12, a formulation used extensively in embryonic neuronal explant and culture studies [34,89,90,91], but which also includes glutamate as well as other excitatory amino acids to enhance cell viability. Indeed, we have found that whole hemisphere explants cultured in neurobasal media, which lacks added excitatory amino acids, show thinner cortical plates and more cell death. However, the presence of amino acid neurotransmitters in the media adds complexity to the results interpretation and further implicates additional factors that may trigger the observed calcium elevations. These additional factors could depolarize the CPN sufficiently to relieve the Mg^2+^ blockade of the NMDA receptor that would then admit calcium.

Collectively, the findings outline a model in which calcium transients are infrequent and intracellular calcium levels are kept low in migrating neurons. During the combined process of dendritic deployment and migration arrest, intracellular calcium levels increase, driven by non-synaptic neurotransmitters released from CRNs. The observed, naturally occurring calcium elevation helps drive dendritic deployment and migration arrest by first increasing growth and branching of the leading process, which prevents further nucleokinesis and soma migration. The rising calcium levels occurring during deployment may contribute to actin cytoskeletal stabilization and enhanced secretory pathway activity that underlies membrane addition for the growing dendrite. In the post-migratory period, baseline calcium levels continue to rise as the nascent dendrite grows into the axonal plexus to receive additional non-synaptic neurotransmitter signaling that drives higher frequency calcium wave activity and further dendritic growth and remodeling. While we did not identify ethanol sensitivity in the CR neuron to CP neuron signaling, it is increasingly evident that many intellectual disabilities [72] and autism-linked genes [92] are expressed during late migration and the early period of dendritic development. Included in this group of risk-alleles that are expressed before synapse formation are genes encoding NMDA receptor subunits, a glycine receptor subunit, and voltage-dependent sodium channels, all proteins which appear to be required for this novel form of CRN to CPN communication.

## 4. Materials and Methods

### 4.1. Mice

Animals were used in compliance with approved protocols by the Institutional Animal Care and Use Committee of SUNY Upstate Medical University. Timed pregnant Swiss Webster (CFW strain from Charles River, Wilmington, MA, USA) were used for these studies, with the day of plug discovery considered E0.

### 4.2. Plasmids

Plasmids containing GCaMP6s (#40753), his-CaMPARI2-F391W-WPRE-SV40 (#101061), and mNaChBac (#60650) were purchased from Addgene (Watertown, MA, USA). These were cloned into a plasmid containing the pCAG promoter [93]. The pCAG- tdTomato encoding plasmid [33] was described previously [3]. The mNaChBac was cloned with a C-terminal FLAG tag. CavMr was generously gifted to us from Dr. Katsumasa Irie (Wakayama Medical University). The CavMr gene was cloned into the pCAG promoter plasmid with an additional C-terminal 6×His tag.

### 4.3. Explants

Whole hemisphere explants were set up as previously described [34,90]. E13 embryos are injected with 2–3 μL of DNA at 0.3–1 mg/mL and electroporated. Explants are cultured medial side down on collagen-coated PTFE filters with 3 μm pores from Corning (Corning, NY, USA), #3494) in DMEM-F12, GlutaMAX (#10565018). The media is supplemented with 1% G5 (#17503012), 1× Penicillin-Streptomycin (#15070063), and 2% B27+ (#A3582801), all from ThermoFisher (Waltham, MA, USA). Explants were maintained in a high oxygen environment (95% O_2_/5% CO_2_) at 37 °C for ~48 h before imaging, experimentation, or fixation.

### 4.4. Calcium Imaging and Analysis

All electroporations were performed with a mix of CAG-GCaMP6s (0.6 mg/mL) and CAG-tdTomato (0.6 mg/mL) on E13. On E15, explants were transferred to the imaging chamber and allowed to thermostabilize under perfusion with warmed, oxygenated media for 10–15 min before imaging (SH7B inline heater, Warner Instruments (Hamden, CT, USA). Calcium imaging was performed using a Thorlabs (Newton, NJ, USA) Accera Series 2-channel multiphoton microscope. A tunable Insight Deep See Multiphoton Ti:Sapphire laser (Spectra Physics, Milpitas, CA, USA) was used to excite GCaMP6s and tdTomato at 930 nm. Two ultrasensitive GaAsP PMTs (Hamamatsu, Bridgewater, NJ, USA) with bandpass 525/50 nm and 605/70 nm filter cubes (Chroma Technology, Bellows Falls, VT, USA) were used for collecting red/green signal. An Olympus XLUMPlanFLN 20×/1.0 water objective (WD = 2 mm) was used for image collection. Timed z-series (2–4 μm z-steps) were collected at intervals varying between 7 s to 10 min for durations between 30 min to 4 h, depending on the experiment.

To quantify GCaMP6s calcium signal, ROIs were placed within the cell of interest on a single z-slice, and the mean pixel intensity was measured on the green (GCaMP6s) and red (td-Tomato) channels, which were individually background corrected and then ratioed. The soma ROI was entirely contained within the cell boundary; the proximal neurite ROI was placed within one cell diameter of the soma, typically at a thick area of the proximal neurite. Neurite tips were measured at or near the end of the leading process of migrating/translocating neurons or the most distal tip of the nascent dendrite in the post-migratory neurons. In most cases, the actual measurement was taken 1–2 μm proximal from the distal tip to measure a thicker portion of the neurite and provide more signal. Distances from the pial surface in this study were measured as a straight line and are, therefore, approximate for cells migrating out of the x, y plane. All measurements were performed in FIJI [94] and the ANOVA analysis was performed between cell types within each ROI category (e.g., soma).

### 4.5. Pharmacology

For GCaMP6s studies, a baseline set of images was collected as discussed. A glutamatergic antagonist cocktail was then added to the perfusion media and allowed to circulate for ~10 min before image collection. For CaMPARI2 studies, explants were incubated with a glutamatergic antagonist cocktail, TTX, environmental insult (ethanol, 400 mg/dL), or control media for 30 min before being challenged with veratridine (100 μM) or control media. All groups were photoconverted and processed for histology, as discussed below.

The antagonist and concentrations employed were: 40 μM bicuculline methochloride (GABAA receptors), 100 μM MK-801 (NMDA receptors), 5 μM MTEP hydrochloride (mGluR5 receptors), 25 μM LY367385 (mGluR1 receptor), 10 μM CNQX (AMPA receptors), 100 μM AP-5 (NMDA receptors), 30 μM strychnine (glycine receptor), and 2 μM tetrodotoxin (voltage-gated sodium channel antagonist). The pan-glutamatergic blockade included: MK-801, LY367385, CNQX, MTEP, and AP-5. The pan-neurotransmitter blockade included: Bicuculline, strychnine, and the pan-glutamatergic cocktail. Veratridine, a voltage-gated sodium channel agonist, was used at 100 μM. Veratridine and all antagonists were from Bio-techne/Tocris Bioscience (Minneapolis, MN, USA). The control media was prepared using the same solvents that were used to dissolve or dilute the various drugs. Specifically, water was used as the solvent for bicuculline methochloride, MTEP hydrochloride, CNQX disodium salt, AP-5, and EtOH. For LY 367385, a solution of 55 mM NaOH was used. DMSO was the solvent for MK-801 and veratridine, while CHCl_3_ was used for strychnine. In each case, the control media contained an equivalent amount of the respective solvent without the drug.

### 4.6. CaMPARI2 Photoconversion and Immunohistochemistry

Photoconversion (PC) was accomplished using a Tresbro UV Resin Curing Light (Tresbro, Seattle, WA, USA) light (20 W output). Explants were exposed to a cycle of 405 nm light consisting of 8 s on and 3 s off for 27 cycles. After photoconversion, explants were screened under a fluorescent dissecting scope. After confirmation of successful photoconversion, explants were fixed and processed as previously described [3]. Briefly, explants were drop-fixed in 4% paraformaldehyde (PFA) for 60 min before being embedded in 10% gelatin. Gelatin blocks containing the explants were then post-fixed in 4% PFA overnight at 4 °C. Blocks were stored in PBS until sectioning.

Explants were sectioned at 100 μm by vibratome and sections were then stored in PBS containing 0.02% sodium azide. Individual sections were then incubated in 200 μL blocking buffer composed of PBS with 0.5% Triton X-100 (Sigma-Aldrich, St. Louis, MO, USA), and 2% Bovine Serum Albumin, (also Sigma-Aldrich). Primary antibodies were made up in blocking buffer at 1:1000 Rat anti-HA (3F10) (SKU #11867423001, Millipore-Sigma, St. Louis, MO, USA) and 1:1,000 Rabbit anti-CaMPARI-Red (4F6) (#Ab01649-23.0, Absolute Antibody, Boston, MA, USA) and 1:500 mouse anti-Ctip1 (#19489, Abcam, Boston, MA, USA). Sections were incubated in primary antibodies overnight at 4 °C.

Sections were washed in PBS for 3 × 15 min before being incubated in secondary antibody solution (AlexaFluor goat anti-rat 555 (#A21434, ThermoFisher), AlexaFluor donkey anti-rabbit (#A31573, Thermofisher), AlexaFluor donkey anti-mouse 647 (#A31571, ThermoFisher). Secondary antibodies were diluted 1:500 in blocking buffer. The nuclear stain Hoechst 33342 (stock 10 mg/mL, Millipore-Sigma) was added to the secondary solution at 1:500. Sections were incubated in the secondary solution for 4 h at RT before being washed 3 × 15 min in PBS. Finally, sections were mounted in Fluoromount-G (#0100-01, SouthernBiotech, Birmingham, AL, USA) on standard slides or 24 well Greiner-Bio One (Kremsmünster, Austria) imaging plates (#662892).

### 4.7. Imaging and Data Analysis

The CaMPARI2 characterization of native calcium signal was performed with a Leica SP8 confocal system in the SUNY Upstate Neuroscience Microscopy Core. Images were collected using the HC PL APO CS2 20×/0.75 objective and with a digital zoom of 2.25, and ~15 μm stacks were acquired at a z-step of 0.65 μm. Images were z-projected and analyzed in FIJI. For these studies, the ROI was placed in the proximal dendrite. The mean intensities of the red and green signals were background corrected and ratioed. Two sections from 5 explants with an average of 48 neurons per explant were analyzed. Sections from CaMPARI2 pharmacology experiments were imaged using a Zeiss (Wetzlar, Germany) LSM 780 laser scanning confocal microscope in the SUNY Upstate Neuroscience Microscopy Core (Syracuse, NY, USA). Images were collected using EC Plan-Neofluar 10×/0.30 objective and a 2.0 digital zoom. Data analysis was done as described above, with an exception: the red and green signal were only collected from neurons in the upper cortical plate, 20 μm below the marginal zone. At least 6 explants and an average of 287 neurons were analyzed for each condition. Higher magnification imaging for morphologic analyses in the ion channel, pan-glutamatergic blockade, and activity block studies was performed using the Zeiss LSM 780 with a C-Apochromatic 40×/1.20 W Korr objective.

### 4.8. Statistics

GraphPad Prism 9.5.1 was used for all statistical analyses. For primarily descriptive statistics (e.g., mean spike amplitude), the mean ± standard deviations (s.d.) are reported. For statistical comparisons, the mean ± standard error of the mean (s.e.m.) is reported. For multiple comparison analysis, we used ordinary one-way ANOVA and Šidák’s multiple comparisons test with a single pooled variance and significance at *p* < 0.05. For pairwise comparison, a one-tailed *t*-test was employed where a clear predicted direction of change was available (e.g., dendrite growth or calcium signal reduction). Alternatively, a two-tailed *t*-test was used for comparisons where the change was not predicted (e.g., calcium dynamics quantification). Significance was set at *p* < 0.05.

## Data Availability

The data presented in this study are available upon request to the corresponding author.
